# Lipopolysaccharide-promoted proliferation of Caco-2 cells is mediated by c-Src induction and ERK activation

**DOI:** 10.7603/s40681-015-0005-x

**Published:** 2015-02-02

**Authors:** Tsung-Yao Lin, Chiung-Wen Fan, Ming-Chei Maa, Tzeng-Horng Leu

**Affiliations:** 1 Institute of Biochemistry, Chung Shan Medical University, 402 Taichung, Taiwan; 2 Institute of Medical Science, China Medical University, 404 Taichung, Taiwan; 3 Department of Pharmacology, College of Medicine, National Cheng Kung University, No. 1, University Road, 701 Tainan, Taiwan; 4 Graduate Institute of Basic Medical Science, No. 91, Hsueh-Shih Road, 404 Taichung, Taiwan

**Keywords:** LPS;, Caco-2;, c-Src;, ERK

## Abstract

As a major component of the cell wall of Gram-negative bacteria, lipopolysaccharide (LPS) can be released into the bloodstream to cause a spectrum of pathophysiological reactions. Despite the fact that colon epithelium cells *in situ* are continuously exposed to LPS, their biological responses as provoked by LPS as well as the underlying mechanisms are poorly defined. In the present study, we observed that LPS directly stimulated growth of Caco-2 cells as well as enhanced the amounts of c-Src, which could be partly attributable to increased *c-src* transcript. Parallel to LPS-induced c-Src expression was FAK activation and ERK activation. Remarkably, activation of ERK and cellular proliferation by LPS could be inhibited by PP2, the specific Src inhibitor, implicating the essential role of c-Src in this process. To our knowledge, this is the first report indicating that LPS can increase cellular growth via upregulation of c-Src in colon epithelial cells.

## 1. Introduction

Epithelia are tissues composed of sheets of similar cells bound closely together that execute different functions such as barrier, adsorption, or secretion. In addition to physically providing the barrier against bacterial infection, epithelia also chemically emit chemotactic signals that attract blood-borne host defense cells to initiate the inflammatory responses after their injury [1]. Due to the production of similar polypeptide arsenals between the epithelial cells and the polymorphonuclear leukocytes, the former are thus thought to be important effectors of innate immunity. Though epithelial cells lining the colon are bathed in bacteria and their products, they remain refractory to the threat from the normal bacterial flora. However, encountering a diverse array of enteroinvasive bacteria, human colon epithelial cells produce not only proinflammatory cytokines, but also an autocrine growthstimulating factor, PGE_2_, to facilitate cellular growth [2]. These observations indicate that colorectal tumors may be formed or activated by exposure to colonic flora or by endotoxemia.

The major membrane component of Gram-negative bacteria, lipopolysaccharide (LPS), is a potent inflammatory stimulus, whose release during growth or lysis of bacteria elicits a diverse array of growth factors, cytokines, and inflammatory mediators [3, 4]. With the help of various LPS binding proteins, LPS induces the activation of responsive cells such as macrophages, endothelial cells and epithelial cells [4]. LPS forms a complex with the LPS binding protein (LPB) in plasma, and the resultant LBP-LPS complexes are recognized by CD14, present either as a GPI-anchored membrane glycoprotein (mCD14), or free as a secreted molecule (sCD14) [3, 4]. Ligation of TLR4-MD2 complex induces by LPS results in activation of multiple signaling proteins including protein tyrosine kinases, members of MAPK family and transcription factors [5].

c-Src is a prototype of a closely related family of nonreceptor tyrosine kinases that function as co-transducers of transmembrane signals emanating from multiple growth factor receptors [6]. To date, proteins such as focal adhesion kinase (FAK), Shc, cortactin, Eps8 and STAT3 have been established as its substrates and their participation in c-Src-mediated physiological activities including proliferation, migration, and cell survival have been established.

Colorectal cancer (CRC) is the most common gastrointestinal cancer. Despite the fact that genetic events leading to its prevalence have been well documented [7], activation of c-Src has also been observed in a majority of studies [8]. Since increased c-Src kinase activity was usually associated with its upregulation, overexpression of c-Src was therefore suggested to play a critical role in colorectal tumorigenesis. Indeed, overexpression of c-Src in murine fibroblasts causes their transformation [9], and anti-sense-mediated downregulation of c-Src in colon adenocarcinoma cells reverts their tumorigenicity [10]. However, to date the precise mechanism underlying c-Src upregulation in CRC is still elusive.

In the present study, we observe that LPS increases the cellular growth of Caco-2 cells. And consistent with this finding is the finding that the expression of c-Src is LPS-inducible and that results in an increase of Src-mediated Shc Tyr-317 phosphorylation, ERK, and FAK activation. Application of PP2 abrogates both LPS-induced ERK activation and cellular proliferation. Notably, this is the first report indicating how the upregulation of c-Src in colon cancer cells is achieved.

## 2. Materials and methods

### 2.1. Cell lines and lysate preparation

The human colon cancer cell line, Caco-2, was utilized in this study. Caco-2 cells were incubated with or without LPS (Sigma). The cells were lysed in ice-cold, modified RIPA buffer as described before [11]. Lysates were centrifuged at 12,000 g for 10 min at 4°C. The protein amount in each lysate was determined by protein assay kit (Bio-Rad).

### 2.2. Antibodies, immunoprecipitation and immunoblotting

For immunoblotting, 100 μg of cell lysates were resolved in an SDS-PAGE and Western immunoblotting with antibody as indicated in the figure legend. Src monoclonal antibodies GD11 and 2-17 were generously provided by Dr. Sarah J. Parsons. Antibodies against actin, amino-terminal FAK (A17), and phosphotyrosine (PY20) were from Santa Cruz Biotechnology (Santa Cruz, California); and the antibody against phosphorylated Tyr- 397 of FAK was from Upstate Biotechnology, Inc. (Lack Placid, NY). The E10 monoclonal antibody recognized phosphorylated Thr-202 and Tyr-204 of ERK1/2 was purchased from New England Biolabs, Inc. (Beverly, MA, USA). The rabbit polyclonal antibodies recognized unphosphorylated ERK2 were purchased from Santa Cruz. Western immunoblotting was performed with antibody of interest and detected by enhanced chemiluminescence (Amersham Biosciences).

### 2.3. Reverse transcription-polymerase chain reaction (RT-PCR)

Total RNA was isolated from Caco-2 cells by utilizing the Trizol reagent (Life Technologies, Inc.) as recommended by the manufacturer. Then the RNA was quantified by spectrophotometer and an equal amount (5 μg) of it was reverse-transcribed into single stranded cDNA in a 50 μl reaction mixture containing reaction buffer (10 mM Tris-HCl [pH 8.3], 15 mM KCl, 0.6 mM MgCl_2_, and 2 mM DTT), 60 pmol of oligo-dT15, 40 U RNase inhibitor, and 200 U of M-MLV reverse transcriptase (Promega). The reaction was carried out at 42°C for 1 h. The single stranded cDNA was diluted 10 times and 10 μl of it was amplified by PCR. The PCR reaction was carried out in a 50-μl mixture containing reaction buffer, 0.2 mM dNTP, 0.2 μM of forward primer and reverse primer for *c-src*, and 2.5 U of Taq polymerase by GeneAmp PCR System 2400 (Applied Biosystems). As an internal standard, a pair of primers for *β-actin* was included at the same time. Unless otherwise indicated, the following program is for *c-src* PCR reaction: the cDNA was denatured for 5 min at 94°C and amplified for 30 cycles under the following conditions: 94°C, 30 sec; 60°C, 40 sec; and 72°C, 1 min, followed by a 5-min elongation step at 72°C. Sequences of primer pairs used were as follows:

**Table 1:** 

*src:*	forward, 5’-CGCTGGCCGGTGGAGTGAC-3’; Reverse, 5’-CCAGCTTGCGGATCTTGTAGTGC-3’;
*β-actin:*	forward: 5’-ATCATGTTTGAGACCTTCAA-3’; Reverse: 5’-CATCTCCTGCTCG AAGTCTA-3’.

PCR products were resolved in a 1% agarose gel and detected by ethidium bormide staining.

**Fig. 1:**
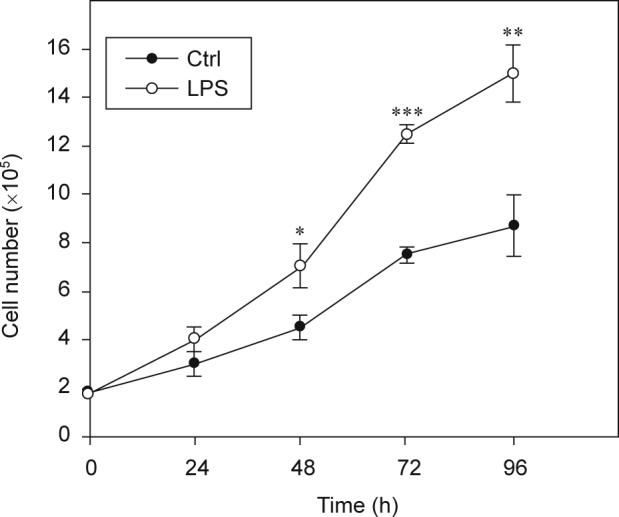
LPS accelerates proliferation in Caco-2 cells. Caco-2 cells (5 × 10^5^) were plated at the beginning. After 18 h, cells were incubated with or without 10 μg/ml LPS for various times as indicated. Total number of control and LPS-treated cells were counted and plotted. The results were shown in means ± SD for three independent experiments performed in triplicate. ^*^ *P* < 0.05; ^**^ *P* < 0.01; ^***^ *P* < 0.001 as compared to its control counterpart.

### 2.4. Statistical analysis

Values given represent the mean ± SD of experiments done in triplicate. Statistical significance was tested by Student’s *t*-test for either paired or unpaired data as appropriate.

## 3. Results

### 3.1. Enhanced cellular proliferation and protein tyrosyl phosphorylation in LPS-treated Caco-2 cells

**Fig. 2:**
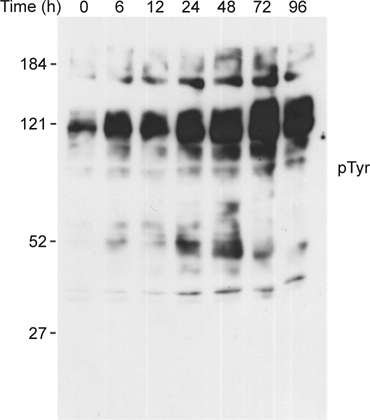
LPS treatment leads to the enhancement of protein tyrosyl phosphorylation in Caco-2 cells. Caco-2 cells were treated with or without 10 μg/ml LPS for various times as indicated. Equal amounts of lysates (100 μg) from each sample were resolved by SDS-PAGE and probed with anti-pTyr Ab. Similar results were repeated three times and the representative was demonstrated. The protein markers (KDa) were shown on the left.

Given that colon contains large numbers of Gram-negative bacteria and colonic epithelial cells *in situ* are continuously exposed to LPS, therefore, the influence of LPS on cellular growth in human colon cancer cells, Caco-2, was analyzed. As shown in Figure 1, compared to non-treated controls, cells exposed to LPS (10 μg/ml) exhibited significant mitogenesis (Figure 1). Thus, these results indicate that LPS can stimulate proliferation in Caco-2 cells. Because tyrosyl phosphorylation plays a critical role in mitogenesis, and to confirm that LPS can augment protein tyrosyl phosphorylation in colon epithelial cells, Caco-2 cells were stimulated with LPS for various time points. As demonstrated in Figure 2, a remarkable time-dependent increase of protein tyrosyl phosphorylation was detected. This finding suggested that LPS could upregulate the expression and/or catalytic activity of a protein tyrosine kinase in colon epithelial cells.

**Fig. 3:**
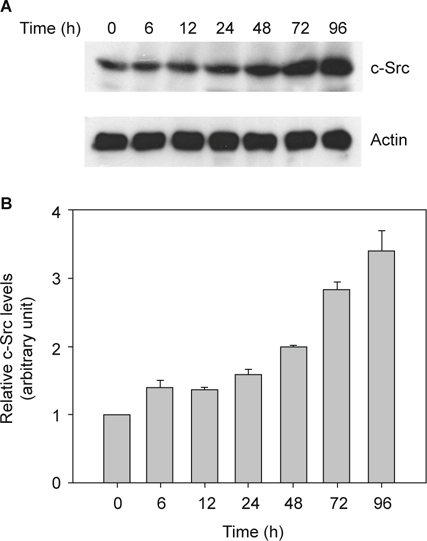
Expression of c-Src is increased in LPS-stimulated Caco-2 cells. (A) Caco-2 cells were treated with or without LPS (10 μg/ml) for various times as indicated. Equal amounts of lysates (100 μg) from each sample were resolved by SDSPAGE and probed with antibodies against c-Src and actin. (B) Densitometric quantification of c-Src expression normalized for actin protein levels; values were means ± SD from three measurements of (A).

**Fig. 4:**
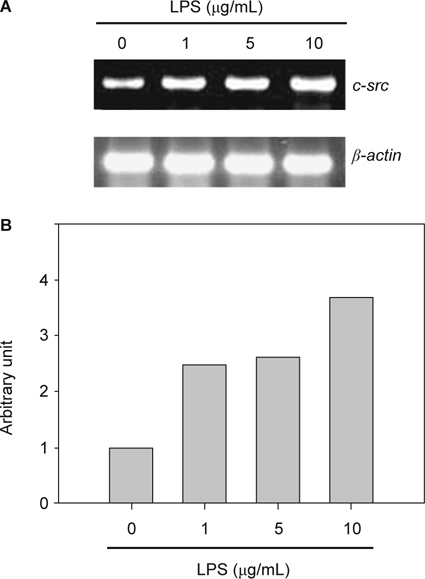
The enhancement of transcript abundance of *c-src* in LPS-treated Caco-2 cells. (A) Caco-2 cells were treated with various concentration of LPS for 96 h. Total RNA was then harvested. Semi-quantitative RT-PCR analysis of *c-src* was performed as described in “Materials and Methods”. *β-actin* was utilized as an internal control for amplification of src efficiency. (B) Densitometric quantification of c-src transcript normalized for *β-actin*; values were mean ± SD from three measurements of (A).

### 3.2. Enhancement of both c-Src protein expression and c-src transcript in LPS-stimulated Caco-2 cells

Previously, we have reported the induction of c-Src in LPSexposed macrophages [11, 12]. To determine whether a similar event could also occur in colon epithelial cells, the expression of c-Src was checked in Caco-2 treated with or without LPS for various time points. As exhibited in Figure 3, compared to the constant expression of actin, a significant time-dependent upregulation of c-Src was observed. To further investigate the effect of LPS on the transcription of *c-src*, we performed the RT-PCR experiments to analyze the transcript abundance of *c-src* in Caco-2 cells stimulated with various concentrations of LPS for 96 h. As demonstrated in Figure 4, after normalization with *actin* transcript, the abundance of *c-src* was greatly increased in response to LPS. These results indicated that LPS could upregulate both the abundance of *c-src* transcript and the protein expression of c-Src in colon epithelial cells.

### 3.3. Increased FAK autophosphorylation and ERK activation in Caco-2 cells exposed to LPS

**Fig. 5:**
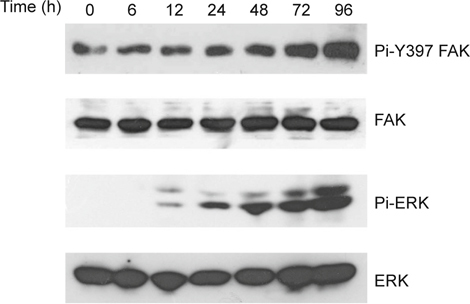
LPS increases Y-397 phosphorylated FAK and ERK activation in Caco-2 cells. Caco-2 cells were incubated with LPS (10 μg/ml) for various times as indicated. Their content of Y-397 phosphorylated FAK, FAK, phosphorylated ERK and ERK was analyzed by direct Western blot analysis, with specific antibody against Pi-Y397 FAK, FAK, phosphorylated ERK (E10), and ERK, respectively.

It is well established that FAK is a substrate for c-Src whose kinase activity, reflected by its autophosphorylation (Pi-Y397 FAK), can be modulated by Src-mediated FAK phosphorylation at Y-576, -577 and 863. Since we have shown that LPS could induce c-Src expression, therefore we wanted to check its total enzymatic activity following LPS exposure. Because the level of Pi-Y397 FAK mirrors the activation of c-Src, thus we chose it as the indicator to assess c-Src kinase activity. Lysates prepared from Caco-2 cells treated with LPS for various time points were analyzed by SDS-PAGE and Western immunoblotted with antibodies against Pi-Y397 FAK and FAK respectively. As shown in Figure 5, while a similar amount of FAK was present in each sample, a remarkable time-dependent increase of FAK activity, measured by the level of Pi-Y397 FAK, was detected; indicating that accompanying the induction of c-Src in LPS-treated Caco-2 cells was the robust increase of its enzymatic activity.

By virtue of c-Src activation, the Raf-MEK-ERK cascade is triggered. Since LPS treatment leads to the enhancement of c-Src expression as well as its total activity, we thereby addressed the point of whether LPS might increase ERK activation by determining the ERK activities in control and LPS-treated Caco-2 cells at various time points. Due to the fact that MEK-mediated ERK phosphorylation on residues Thr-202 and Tyr-204 increased the enzymatic activity of ERK, we therefore applied the monoclonal antibody specifically recognized these phosphorylated residues of ERK in Western immunoblotting. As shown in Figure 5, a significant time-dependent increase of phosphorylated ERK was detected in LPS-exposed Caco-2 cells as compared to the control when the expression of ERK in these cells was normalized.

### 3.4. PP2 abolished LPS-mediated ERK activation and LPS-induced proliferation in Caco-2 cells

**Fig. 6:**
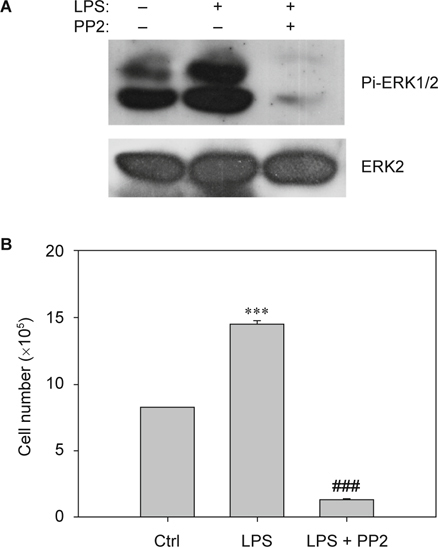
PP2 suppresses the LPS-increased ERK activation and proliferation in Caco-2 cells. Caco-2 cells were pretreated with PP2 (10 μM) for 20 min and then stimulated with or without LPS (10 μg/ml) for 96 h. (A) The content of phosphorylated ERK and ERK were determined by direct Western blot analysis with specific antibody against phosphorylated ERK (E10), and ERK, respectively. (B) Caco-2 cells (5 × 10^5^) were handled as described in (A). After a 96-h incubation, cells of each treatment were harvested and counted. The results are shown as means ± SD of triplicate experiments. ^***^ *P* < 0.001 as compared to Ctrl. ^###^ *P* < 0.001 as compared to LPS-treated cells. Similar results were obtained at least twice.

Mounting evidence reveals that ERK activation is critical for cell proliferation. The increased ERK activity accompanied with LPS-induced c-Src expression indicated that LPS could promote cellular growth via its induction of c-Src. To prove this hypothesis, LPS-elicited ERK activation and mitogenesis were determined in Caco-2 cells pretreated with or without PP2, the inhibitor for Src family members. Interestingly, PP2 not only abrogated LPS-increased ERK activation, but it also suppressed LPS-mediated cellular growth (Figure 6). These results can be taken as evidence to support the role of c-Src induction in LPSmediated Caco-2 proliferation.

## 4. Discussion

Our study proposes a pivotal role for LPS in promoting cellular growth via the induction of c-Src in a human colon carcinoma cell line (Caco-2). Accompanied with the enhancement of c-Src is its augmented activity, which provokes the activation of FAK and ERK. Remarkably, application of PP2 not only abrogates ERK activation, but it also inhibits cell proliferation in LPS-stimulated Caco-2 cells. Thus, the concomitant c-Src induction, ERK activation, and enhanced mitogenesis in colon epithelial cells in response to LPS offer a new perspective on the contribution of LPS to colon carcinoma progression.

Induction of c-Src by LPS has previously been reported in macrophages [11, 12] that retain TLR4, the receptor for LPS. Here, a similar phenomenon is also observed in Caco-2, the colon epithelial cells. Though epithelial cells of the gastrointestinal tract were initially described to lack the expression of TLR4, current literature has provided evidence supporting its expression in a spectrum of intestinal colonocytes including Caco-2 [13, 14]. Thus, like macrophages, colon epithelial cells could also be considered as LPS-responsive. Indeed, LPS was reported to induce the proinflammatory cytokine IL-8 in HT29 and SW620 epithelial cells [15, 16] as well as prostaglandin E_2_ (PGE_2_) in human adenocarcinoma CE-1 cells [17].

ERK appears to be an important signaling molecule in LPSinitiated activation of intestinal epithelial cells (IECs). In contrast to its participation in LPS-induced heat shock protein 25 (HSP25) in young adult mouse colon (YAMC) cells [2], ERK activation mediated by c-Src induction renders LPS-exposed Caco-2 cells more mitogenic. It is noteworthy that in addition to ERK, LPSelicited proliferation can also be attributable to the generation of PGE_2_, which can act as an autocrine growth-stimulating factor [17]

Aberrant expression of c-Src has been closely linked to initiation and progression of human CRC [18]. However, to date, no underlying mechanism has been proposed to elucidate the upregulation of c-Src in IEC. Given that IECs are continuously exposed to high concentrations of LPS in the colonic fluid, LPS-induced c-Src expression therefore provides an immediate explanation for c-Src upregulation in CRC. Based on our studies in both macrophages and epithelial cells, LPS-elicited c-Src expression seems to be a “universal phenomenon”. While overexpression of c-Src in colon epithelial cells may cause a catastrophe, chemicals that attenuate its expression may provide remedies. Indeed, sodium butyrate, the major fermentation product of dietary fiber, appears to be an ideal choice for this purpose. In a separate, recent study, we have demonstrated that by virtue of reducing *c-src* transcript as well as c-Src protein expression, butyrate could effectively arrest the growth of neoplasmic colonocytes [19].

In summary, our demonstration of ERK activation accompanied with augmented c-Src expression plays an essential role in LPS-mediated proliferation in IECs. Notably, *c-src* was not among the LPS-responsive genes identified by microarrays [20, 21]. Considering the role of *c-src* in transformation and its LPSinducible characteristic, our findings might supplement and extend the current understanding of the mechanisms by which LPS exerts its effects in the development of human cancers.

### Acknowledgements

We thank Dr. Sally Parsons for providing Src antibody (GD11). This work was supported by National Science Council grants to M.-C.M (NSC92-2311-B-040-004) and T.-H.L (NSC90-2311-B- 006-007), as well as grants by the NHRI (NHRI-EX-91-8932SL) and the MOE Program for Promoting Academic Excellence of Universities (91-B-FA09-1-4) to T.-H.L.

### Declaration of interest

The authors declare no conflicts of interest for this work.

## References

[1] Epithelia Ganz T. (2002). not just physical barriers. Proc Natl Acad Sci USA.

[2] Kojima K, Musch MW, Ropeleski MJ, Boone DL, Ma A, Chang EB. *Escherichia coli* LPS induces heat shock protein 25 in intestinal epithelial cells through MAP kinase activation. Am J Physiol Gastrointest Liver Physiol 2004; 286: G645–52. 10.1152/ajpgi.00080.2003 14630641

[3] Holst O, Ulmer AJ, Brade H, Flad HD, Rietschel ET. (1996). Biochemistry and cell biology of bacterial endotoxins. FEMS Immunol Med Mic.

[4] Tobias PS, Tapping RI, Gegner JA. (1999). Endotoxin interactions with lipopolysaccharide-responsive cells. Clin Infect Dis.

[5] Rosenberger CM, Finlay BB. (2003). Phagocyte sabotage: disruption of macrophage signalling by bacterial pathogens. Nature Reviews Mol Cell Biol.

[6] Leu TH and Maa MC. Functional implication of the interaction between EGF and c-Src. Front Biosci 2003, 8: s28–38. 10.2741/980 12456372

[7] Kinzler KW, Vogelstein B. (1996). Lessons from hereditary colorectal cancer. Cell.

[8] Dehm S, Senger M-A, Bonham K. (2001). SRC transcriptional activation in a subset of human colon cancer cell lines. FEBS Letters.

[9] Lin PH, Shenoy S, Galitski T, Shalloway D. (1995). Transformation of mouse cells by wild-type mouse c-Src. Oncogene.

[10] Ellis LM, Staley CA, Liu W, Fleming RY, Parikh NU, Bucana CD (1998). Down-regulation of vascular endothelial growth factor in a human colon carcinoma cell line transfected with an antisense expression vector specific for c-src. J Biol Chem.

[11] Leu TH, Charoenfuprasert S, Yen CK, Fan CW, Maa MC. (2006). Lipopolysaccharide induced c-Src expression plays a role in nitric oxide and TNFα secretion in macrophages. Mol Immunol.

[12] Maa MC, Chang MY, Li J, Li YY, Hsieh MY, Yang CJ (2011). The iNOS/Src/FAK axis is critical in Toll-like receptor-mediated cell motility in macrophages. Biochim Biophys Acta.

[13] Cario E, Rosenberg IM, Brandwein SL, Beck PL, Reinecker HC, Podolsky DK. (2000). Lipopolysaccharide activates distinct signaling pathways in intestinal epithelial cell lines expressing toll-like receptors. J Immuno.

[14] Backhed F, Hornef M. (2003). Toll-like receptor 4-mediated signaling by epithelial surfaces: necessity or threat?. Microbes Infect.

[15] Pugin J, Schurer-Maly CC, Leturcq D, Moriarty A, Ulevitch RJ, Tobias PS. (1993). Lipopolysaccharide activation of human endothelial and epithelial cells is mediated by lipopolysaccharide-binding protein and soluble CD14. Proc Natl Acad Sci USA.

[16] Eckmann L, Jung HC, Schurer-Maly C, Panja A, Kagnoff MF. (1993). Differential cytokine expression by human intestinal epithelial cell lines: regulated expression of interleukin 8. Gastroenterology.

[17] Kojima M, Morisaki T, Izuhara K, Uchiyama A, Matsunari Y, Katano M (2000). Lipopolysaccharide increases cyclo-oxygenase-2 expression in a colon carcinoma cell line through nuclear factor-κB activation. Oncogene.

[18] Irby RB, Yeatman T. (2000). Role of Src expression and activation in human cancer. Oncogene.

[19] Lee JC, Maa MC, Yu HS, Wang JH, Yen CK, Wang ST (2005). Butyrate regulates the expression of c-Src and focal adhesion kinase and inhibits cell invasion of human colon canceer cells. Mol Carcinogen.

[20] Bjorkbacka H, Fitzgerald KA, Huet F, Li X, Gregory JA, Lee MA (2004). The induction of macrophage gene expression by LPS predominantly utilizes Myd88-independent signaling cascades. Physiol Genomics.

[21] Jeyaseelan S, Chu HW, Young SK, Worthen GS. (2004). Transcriptional profiling of lipopolysaccharide-induced acute lung injury. Infect Immun.

